# Supplementary material for: Thermal noise and optomechanical features in the emission of a membrane-coupled compound cavity laser diode
Source: Sci Rep. 2016 Aug 19;6:31489. doi: 10.1038/srep31489 (PMC4990904; doi:10.1038/srep31489)
Supplement: Supplementary Information [file srep31489-s1.pdf]

Supplementary Information: Thermal noise and optomechanical features in the emission of a membrane-coupled compound cavity laser diode

Lorenzo Baldacci[1,2,+,*], **Alessandro Pitanti**[1,+], **Luca Masini**[1], **Andrea Arcangeli**[1], **Francesco Colangelo**[1], **Daniel Navarro-Urrios**[1,3], **and Alessandro Tredicucci**[4]

[1]NEST, CNR Istituto Nanoscienze and Scuola Normale Superiore, Piazza San Silvestro 12, 56127, Pisa (Italy)
[2]Institute of Life Sciences, Scuola Superiore Sant'Anna, Piazza Martiri della Libertà 33, 56127, Pisa (Italy)
[3]Catalan Institute of Nanoscience and Nanotechnology (ICN2), CSIS and the Barcelona Institute of Science and Technology, Campus UAB, Bellaterra, 08193, Barcelona (Spain)
[4]NEST, CNR Istituto Nanoscienze and Dipartimento di Fisica, Università di Pisa, Largo Pontecorvo 3, 56127, Pisa (Italy)
[*]l.baldacci@sssup.it
[+]these authors contributed equally to this work

1 Spectral properties and sensitivity of the Littrow cavity diode laser coupled to a movable mirror

In the main article we measured the mechanical motion of a silicon nitride trampoline membrane by employing it as a mirror of a compound cavity laser made by a laser diode, a Littrow grating, and the membrane itself. The optical feedback provided by the membrane modifies the emission properties of the laser according to the membrane position, and this property was exploited for sensing the membrane motion down to its thermal noise. At the same time we modeled the optical properties of our compund cavity on the basis of an analytical treatment[1,2] to theoretically predict the displacement sensitivity of our experimental setup. The model, though analytical, is a bit harsh to develop; for the sake of readability, we reported only the main results in the text, leaving the details to this compendium. In this section we will show how to estimate the spectral properties and the displacement sensitivity of the laser system shown in Fig. S1. The procedure here adopted is in large part derived from the works of Detoma and Tromborg.[1,2] We chose to follow this analytical procedure because it gives some insights on how the various parameters act on the spectral and sensing characteristics of the system; despite its simplicity, it includes modal stability analysis and predicts many spectral properties typical of laser systems with external optical feedback, such as the double-peaked power spectrum when the lasing mode becomes unstable in moderate to strong feedback regime.[1] On the other hand, due to the complexity of the physical effects here investigated our model does not allow for highly accurate predictions, although qualitative guidelines on the system behavior can be extracted. This simple treatment is made at the expense of some approximations; mainly, the dynamics of the photon build-up is considered to be dominated by one single mode, and our compound cavity is assumed to host the maximum gain laser mode as the dominant mode at the beginning.[3,4] After the mode with lowest threshold gain is established, the system evolves following the analytical model as the membrane is changed in position. The laser gain is assumed to be spatially uniform, with two phenomenological parameters, the differential gain g_n and the gain saturation factor g_p, accounting for gain enhancement due to the variations of both carrier density and photon number. The

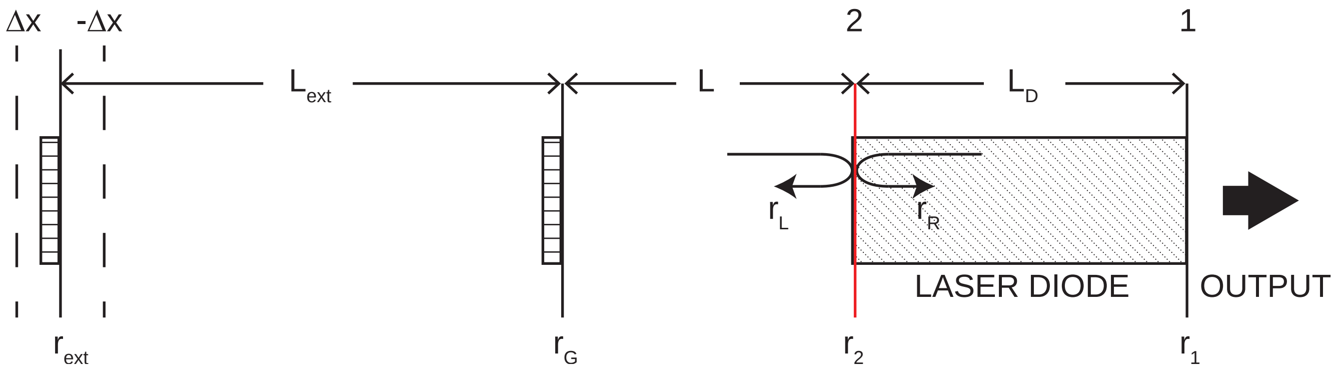

Figure S1. Sketch of the system. The laser diode is characterized by its geometrical length L_D, its volume V_c, the optical gain $g(N,P) = g_{th} + g_n(N - N_{th}) + g_p P$, and the reflectivities r_1 and r_2 relative to its facets called 1 and 2 in figure. Outside the active region, a grating with dispersive reflectvity r_G is placed at a distance L from the diode facet 2, while a movable mirror with reflectivity r_{ext} is placed at a distance L_{ext} from the grating surface. The red line indicates the reference plane chosen to define the reflection coefficients r_R and r_L, which link the electric field of the system traveling in the right direction to the one traveling in the left direction, according to equations (S1a) and (S1b). The reference plane is placed immediately after the right-hand face of laser facet 2, so that r_R is the result of the three-mirror cavity made by r_2, r_G and r_{ext}, while r_L includes field amplification effects within the active region.

analysis presented here involves many parameters, whose numerical value we report in Table S1, including a brief description of the physical quantity they represent.

In the first paragraph we will give the oscillation conditions for the compound cavity modes, and try to interpret them in view of the membrane feedback. Thanks to these conditions we will be able to find a relation linking the change in photon number to the change in the membrane position. The second paragraph will treat the fluctuations in the laser field. This is useful for two reasons: define the range of stability of the laser mode and the range of displacements detectable before instabilities occur; estimate the Relative Intensity Noise, and the fundamental imprecision related to the displacement measurement. In the third paragraph, the conditions found in the first two paragraphs will be finally applied to estimate the displacement sensitivity of our system.

1.1 Oscillation condition and system modes

As a first step, we are going to find the oscillation condition in the frequency domain for the compound cavity electromagnetic modes, assuming no photon noise. We still keep the sketch of Fig. S1 as our toy model, and express the electric field in a traveling wave picture. We take the plane immediately after the right-hand face of laser facet 2 as the reference plane (see the red line in the figure), then we define $\mathscr{E}^+$ as the field traveling in the left direction, and $\mathscr{E}^-$ as the one traveling in the right direction. They are linked through the coefficients r_L and r_R:

$$\mathscr{E}^+ = r_L \mathscr{E}^-, \tag{S1a}$$
$$\mathscr{E}^- = r_R \mathscr{E}^+. \tag{S1b}$$

By calling $\mathscr{E}^+ = \mathscr{E}$ and making simple substitutions we obtain

$$(1 - r_L r_R)\mathscr{E} = 0; \tag{S2}$$

the only solutions with non-vanishing $\mathscr{E}$ are given by

$$r_L r_R = 1, \tag{S3}$$

which is the oscillation condition for the compound cavity modes. It is interesting to expand r_R and r_L using the physical features of the system. r_R can be seen as the effective reflectivity of the three combined mirrors, the diode facet 2 with reflectivity r_2, the Littrow grating with r_G and the membrane with r_{ext}. By applying recursively the formula for the reflectivity of a double mirror cavity, we get:

$$r_R = \frac{r_2 + r_e e^{-i\omega\tau}}{1 + r_2 r_e e^{-i\omega\tau}}, \tag{S4}$$

with

$$r_e = \frac{r_G + r_{ext} e^{-i\omega\tau_{ext}}}{1 + r_G^* r_{ext} e^{-i\omega\tau_{ext}}}, \tag{S5}$$

where ω is the angular frequency of the lasing mode, $\tau = 2L/c_0$ is the radiation roundtrip time between the facet 2 and the grating, and $\tau_{ext} = 2L_{ext}/c_0$ is the one between the grating and the membrane. r_L results from the propagation of the field throughout the diode cavity and can be expressed as

$$r_L = r_1 e^{-2iL_D k(\omega,N,P)}, \tag{S6}$$

where r_1 is the reflectivity at the diode facet 1, L_D is the active region length, N and P are the laser carrier density and photon number, while

$$k(\omega,N,P) = \frac{\omega}{c_0} n + \frac{1}{2} i(g(\omega,N,P) - a_{in}). \tag{S7}$$

We simplify the analysis by assuming the group index n to be constant, as well as the internal losses a_{in} and the gain dispersion with frequency. In addition to that, the cavity mode oscillating at threshold is assumed to be the one which sees the highest right reflectivity, called r_m, and thus requires the lowest threshold gain $g_0(\tau_{ext}) = a_{in} - 1/L_D \log\left(|r1||r_m(\tau_{ext})|\right)$. Within this approximations we can express g as

$$g(\tau_{ext},N,P) = g_0(\tau_{ext}) + (1 + i\alpha) g_n \Delta N + g_p P, \tag{S8}$$

where $\Delta N = N - N_{th}$. Note that $|r_m|$ slightly depends on the position of the membrane, as can be seen from equation (S4), and in our case it ranges from 0.9009 to 0.9011. The magnitude of this modulation depends on the ratio between $\Delta\omega_g$ and the total external cavity free spectral range. As an example, if these two have the same value, this modulation would not exceed 3%. In our case it can be seen as a slight perturbation in the parameters contributing to the oscillation condition. P can be defined as a function of ΔN after the rate equation for the system is defined:

$$\frac{\mathrm{d}N}{\mathrm{d}t} = \frac{I}{qV_c} - \frac{N}{\tau_s} - (\gamma + V_c G_N \Delta N + G_P P)\frac{P}{V_c}, \tag{S9}$$

where $\gamma = v_g g_0$, $G_N = v_g g_n / V_c$, $G_P = v_g g_p$, with v_g group velocity in the active region. As we look for the stationary solutions, this equation can be easily solved for P by putting $\mathrm{d}N/\mathrm{d}t = 0$, and we find that

$$P(\Delta N) = \frac{1}{2G_p}\left(-(\gamma + V_c G_n \Delta N) + \sqrt{(\gamma + V_c G_n \Delta N)^2 - 4\left(\frac{V_c \Delta N}{\tau_s} - \frac{(I - I_0)}{q}\right) G_p}\right), \tag{S10}$$

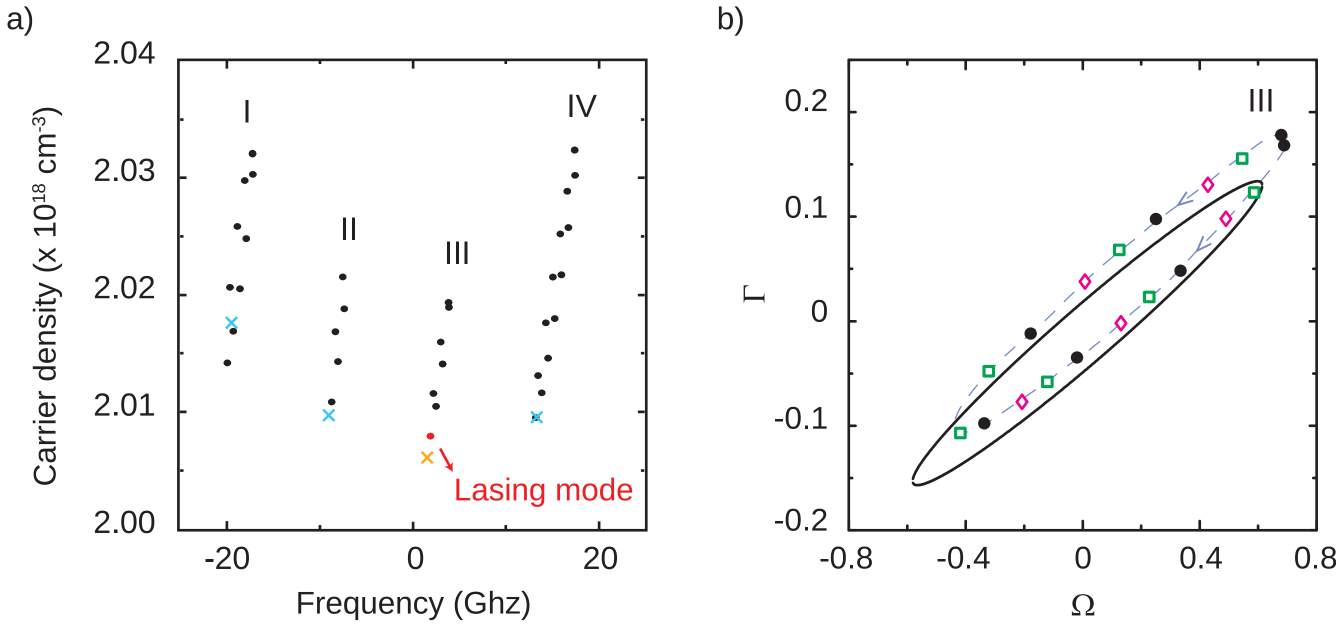

Figure S2. a) Oscillating modes for the laser compound cavity found by solving equation (S3) with the membrane (solid circles) and without it (crosses). The red circle and orange cross indicate the respective lasing modes. The solutions with the membrane have been obtained for $L_{ext} = 20\,\mathrm{cm} + 200\,\mathrm{nm}$, and they can be grouped into four subsets, labeled with roman numbers from I to IV. b) Set III represented in the (Ω, Γ) plane for for three different positions of the membrane, $L_{ext} = 20$ cm (purple diamonds), $L_{ext} = 20\,\mathrm{cm} + 200\,\mathrm{nm}$ (black circles), $L_{ext} = 20\,\mathrm{cm} + 345\,\mathrm{nm}$ (green squares). If we considered the feedback of the membrane as a first order perturbation to the Littrow laser cavity lasing regime, we would find the oscillating solutions lying on the solid black curve, which is the solution of equation (S16). The approximated curve fits well the oscillating modes of the system, and the feedback strength can be estimated according to equation (S17).

where the plus sign has been chosen by imposing $\Delta N = \frac{(I-I_0)\tau_s}{qV_c}$ when $P = 0$.
At this point we have all the elements to solve equation (S3) for (ω, N) and finally find the oscillating modes for the compound cavity.
As an example, using the parameters of Table S1 and a membrane displacement around the equilibrium position of 200 nm we obtain the set of points shown in Fig. S2a. We assume that the lasing mode is the one with the lowest threshold (red circle in the *III* set in figure). Within the same figure, the crosses refer to the Littrow External Cavity Diode Laser (ECDL) oscillating modes with the same parameters and with no external membrane feedback; this time the feedback is given by the Littrow grating itself on the diode cavity.[2]
If we consider the feedback effect of the membrane as a first order perturbation on the Littrow ECDL lasing mode, we can treat the system within a Lang-Kobayashi formalism to estimate the feedback strength brought by the membrane. In order to verify whether this treatment is a good approximation, we look for an approximated analytical solution in the (ω, N) plane describing the oscillating solutions of our system.[2,5] If the numerical solutions of equation (S3) are fitted well for different L_{ext}, then we can use the analytical curve to obtain the feedback strength brought by our membrane. When the feedback is turned on, we see that the oscillating modes of the Littrow ECDL are turned into solution subsets like the ones indicated with roman numbers in Fig. S2a. In particular, we restrict our analysis to the sole Littrow ECDL

lasing mode, with angular frequency ω_1 and carrier density N_1, indicated by the orange cross in Fig. S2a. By neglecting r_2 and g_p and considering the grating reflectivity as constant, $r_G = r_g$ it reads as

$$r_g r_1 e^{-i\omega_1\tau - 2ik_1 L_D} = 1\,, \tag{S11}$$

with k_1 defined according to equations (S7) and (S8). When the feedback is turned on multiple solutions are found in place of this one. As r_{ext} is smaller than r_g, we expect the new solutions to lie along a closed curve. Then we combine equation (S11) with equations (S3) and (S4) to obtain

$$e^{i\xi} = \frac{1}{r_g}\frac{r_g + r_{ext}e^{-i\omega\tau_{ext}}}{1 + r_g r_{ext}e^{-i\omega\tau_{ext}}}\,, \tag{S12}$$

with

$$i\xi = 2iL_D(k - k_1) = i(\tau_{in} + \tau)(\omega - \omega_1) - (1/2)(1 + i\alpha)\tau_{in}v_g g_n V_c(N - N_1) - \log r_g/|r_m|\,. \tag{S13}$$

We can collect the phase term dependent by τ_{ext},

$$e^{i\omega\tau_{ext}}(e^{i\xi} - 1) = \frac{r_{ext}}{r_g}(1 - r_g^2 e^{i\xi})\,, \tag{S14}$$

then expand to the first order the exponential in ξ and take the square modulus by both sides:

$$|i\xi|^2 = \frac{r_{ext}^2}{r_g^2}|1 - r_g^2 - ir_g\xi|^2\,. \tag{S15}$$

This equation is independent from τ_{ext}, therefore it is verified by the oscillating modes of our system at different membrane positions. By definining $\kappa = (r_{ext}/r_g)(1 - r_g^2)$ (which resembles the typical feedback parameter) and the normalized variables $\Gamma = 1/2\tau_{in}v_g g_n V_c(N - N_0) + \log r_g/|r_m|$ and $\Omega = (\tau_{in} + \tau)(\omega - \omega_0) + \alpha\log r_g/|r_m|$, the solutions of equation (S15) describe an ellipse in the (Γ, Ω) plane:

$$\Gamma = \frac{\alpha(\Omega - r_g^2\kappa^2) \pm \sqrt{[1 - r_{ext}^2 + \alpha^2(1 + r_{ext}^2 r_g^2)]\kappa^2 + (1 - r_{ext}^2)r_{ext}r_g\kappa\Omega - (1 - r_{ext}^2)\Omega}}{1 - r_{ext}^2 + \alpha^2}\,. \tag{S16}$$

The resulting curve can be used to fit the set of oscillating modes reported in Fig. S2a. By solving equation (S3) for different L_{ext} we found that the solutions in subset III actually evolve along a closed curve which resembles well that one from equation (S16). In Fig. S2b we give a graphical proof by plotting equation (S16) as a solid black curve in the (Ω, Γ) plane, and the solutions of equation (S3) calculated for three different L_{ext}, 20 cm (purple diamonds), 20 cm + 200 nm (black circles) and 20 cm + 345 nm (green squares). As the position of the membrane is moved forward from $L_{ext} = 20$ cm, the purple diamond solutions move toward smaller Ω following the blue dashed line; at $L_{ext} = 20$ cm + 200 nm the solutions have turned into the black circles, while two new solutions arose at higher Ω. Moving on to $L_{ext} = 20$ cm + 345 nm, the solutions keep moving toward smaller Ω, and the leftmost solution reaches the minimum Γ.

Within the same approximations, the feedback strength of the membrane can be written as:

$$C = \kappa\frac{\tau_{ext}}{\tau_{in} + \tau}\sqrt{1 + \alpha^2} = 8.99; \tag{S17}$$

If this approximation is consistent, different definitions of C must be consistent; for example, C can be also estimated by evaluating the total frequency range of stability Δf_s, through the equation[6]

$$2\pi\Delta f_s \tau_{ext} = 2C\,. \tag{S18}$$

The full solution of equation (S3) with our set of parameters gives a stability range of 2.17 GHz (the stability analysis will be treated in subsection 1.2). Δf_s is spanned completely by varying L_{ext} of about 1654 nm. Therefore, τ_{ext} can be assumed to be constant within 0.001%, which is acceptable for the purpose of this estimate. From equation (S18) the feedback parameter results $C = 9.08$, which is in good agreement with the one predicted from equation (S17).

In conclusion, the oscillating modes of a system like the one sketched in Fig. S1 can be found by solving equation (S3), which can be expanded as a function of the laser features, such as the gain, and the membrane position, which basically affects r_R. The contribution of the membrane to the oscillation condition can be seen as originating from its moderate feedback action onto the Littrow ECDL; for each solution of this one, the complete system hosts a group of solutions, lying to a reasonable approximation onto an ellipse described by equation (S16).

1.2 Mode stability and RIN

Once we have defined the lasing mode of the compound cavity, it is important to ascertain its stability when the membrane vibrates around its initial position. In this case, as soon as it is found to be stable, the lasing mode varies continuously, following the membrane vibrations. Given a position of the membrane, the lasing mode is defined by the set of parameters $(\omega_s, \Delta N_s, P_s)$ according to equation (S3). Within a small signal analysis treatment, this mode is stable if small fluctuations are damped, thus the mode is called back to the initial solution. We may separate the field as the product between a term oscillating at the laser optical frequency, called ω_s, and a slowly varying term. Therefore, in the time domain the field is expressed by

$$E_{laser}(t) = E(t)e^{i\omega_s t}\,. \tag{S19}$$

In this small signal treatment we focus on the field envelope $E(t)$. Its time domain expression can be found by performing the inverse Fourier transform of the oscillation condition, this time including the spontaneous emission noise $\mathscr{F}_{sp}(\omega)$:[1]

$$(1 - r_L r_R)\mathscr{E}(\omega) = \mathscr{F}_{sp}(\omega)\,; \tag{S20}$$

because we are interested in the slowly varying fluctuations, the rapidly oscillating terms can be eliminated by averaging over one diode cavity round-trip time τ_{in} to find

$$E(t) = \exp\left\{\frac{1}{\tau_{in}}\int_{t-\tau_{in}}^{t}[L_D(1+i\alpha)g_n\Delta N(t) + L_D g_p P(t)]dt'\right\}[r(t) * E(t)]_{t-\tau_{in}} + F_{sp}(t)\,. \tag{S21}$$

$F_{sp}(t)$ is the spontaneous emission noise in the time domain, $r(t)$ is the inverse Fourier transform of $r_R r_1 \exp(g_0 L_D)$, while $[r(t) * E(t)]_{t-\tau_{in}}$ is the time domain convolution evaluated at $t - \tau_{in}$. ΔN and P are defined as $\Delta N(t) = \Delta N_s + \delta N(t)$ and $P(t) = P_s + \delta P(t)$, where $\delta N(t)$ and $\delta P(t)$ are their fluctuations. If the position of the membrane changes much more slowly than all other terms in equation (S21), its contribution to $E(t)$ can be decoupled considering a second envelope. In this case oscillating solutions, stability and noise properties of $E(t)$ can be calculated for each static membrane position, defined by $L_{ext,0}$

plus a static displacement Δx_{theo}. Now, given the lasing mode with $(\omega_s, \Delta N_s, P_s, E_s)$, we perform the small signal analysis in the frequency domain. We define the Fourier transform of $E(t)$ as

$$\int_{-\infty}^{\infty} E(t) e^{-i\tilde{\omega}t} dt = 2\pi E_s \delta(\tilde{\omega}) + \delta E(\tilde{\omega}), \tag{S22}$$

with $\tilde{\omega} = \omega - \omega_s$, $\delta(\tilde{\omega})$ is the Dirac delta and E_s is the lasing mode amplitude. Equation (S21) can be directly transformed and expanded as a function of $\delta E(\tilde{\omega})$, by defining the carrier density and photon number fluctuations:

$$\delta P(\tilde{\omega}) = E_s(\delta E(\tilde{\omega}) + \delta E^*(-\tilde{\omega}^*)), \tag{S23a}$$

$$\delta N(\tilde{\omega}) = \frac{1}{V_c(i\tilde{\omega} + \Gamma_N)} \left[\frac{F_I(\tilde{\omega})}{q} - (\gamma + V_c G_N \Delta N_s + G_P P_s)\delta P(\tilde{\omega}) \right], \tag{S23b}$$

where the complex extension of $\tilde{\omega}$ has been considered. In the first equation we assumed a normalization such that $P_s = |E_s|^2$. The second equation needs a brief description. We define $N(t) = N_s + \delta N(t)$ and expand equation (S9) to the first order in $\delta N(t)$ and $\delta P(t)$:

$$\frac{d\delta N(t)}{dt} = \frac{F_I(t)}{qV_c} - \frac{\delta N(t)}{\tau_s} - (\gamma + V_c G_N \Delta N_s + G_P P_s)\frac{\delta P(t)}{V_c} - G_N P_s \delta N(t). \tag{S24}$$

This equation is then Fourier transformed to obtain equation (S23b). With respect to the treatment proposed by Detoma,[1] we decided to add a noise term $F_I(t)$ due to the bias current, because its contribution to the spectral properties could be relevant when the laser is driven well above threshold.[7,8] Note that $F_{sp}(t)$ and $F_I(t)$ are related to the single occurrence of random processes. Also this quantities are averaged over τ_{in} just like the gain, thus being smoothed in the time-domain; unlike their rigorous mathematical description, these quantities are differentiable and their Fourier transform can be defined. This is important in the following treatment because we want to find the spectral properties of the field fluctuations in our system. The carriers velocity is modeled to suffer damped Brownian diffusion,[7,8] thus $F_I(t)$ can be defined according to the Ornstein-Uhlenbeck model:

$$\langle F_I(t) F_I(t') \rangle = \sigma_I^2 \exp\left\{ \Gamma_I |t - t'| \right\}, \tag{S25}$$

where Γ_I is the damping rate of the process, and the angled brackets $\langle\rangle$ refer to the average over the statistical ensemble; within the stationary regime the probability density function is a simple Gaussian $dP(F_I) = \sqrt{\Gamma_I/(\pi\sigma_I^2)} \exp[-\Gamma_I F_I^2/\sigma_I^2]$. The stationarity of Ornstein-Uhlenbeck processes also ensures the validity of Wiener-Khinchin theorem for the power spectral density of $F_I(\tilde{\omega})$ (we will use it afterward for calculating the relative intensity noise of our laser system):

$$\langle F_I(\tilde{\omega}) F_I^*(\tilde{\omega}) \rangle = \frac{2\sigma_I^2 \Gamma_I}{\tilde{\omega}^2 + \Gamma_I^2} \tag{S26}$$

Going back to equation (S21), its Fourier transform can be carried out straightforwardly by applying the convolution theorem between the exponential functon and $r(t) * E(t)$, and the simple properties related to the Fourier transform of an integral function. We thus find

$$[1 - \mathscr{G}(\tilde{\omega}) + B(\tilde{\omega})]\, \delta E(\tilde{\omega}) + B(\tilde{\omega}) \delta E^*(-\tilde{\omega}^*) = F(\tilde{\omega}), \tag{S27}$$

where we introduced the following parameters:

$$\mathscr{G} = 1 - \frac{r_R(\omega_s + \tilde{\omega})}{r_R(\omega_s)} e^{-i\tilde{\omega}\tau_{in}}, \tag{S28a}$$

$$B(\tilde{\omega}) = \frac{1 - e^{-i\tilde{\omega}\tau_{in}}}{2i\tilde{\omega}} \left[(1 + i\alpha) \left(\frac{G_N \gamma P_s + G_N^2 V_c \Delta N_s P_s + G_N G_P P_s^2}{i\tilde{\omega} + \Gamma_N} \right) - G_P P \right], \tag{S28b}$$

$$F(\tilde{\omega}) = F_{sp}(\tilde{\omega}) + \frac{1 - e^{-i\tilde{\omega}\tau_{in}}}{2i\tilde{\omega}} (1 + i\alpha) \frac{G_N E_s}{q} \frac{F_I(\tilde{\omega})}{i\tilde{\omega} + \Gamma_N}. \tag{S28c}$$

$B(\tilde{\omega})$ encloses the coupling strength between modes at frequency $\omega_s + \tilde{\omega}$ and the ones at $\omega_s - \tilde{\omega}^*$, which derives from fluctuations in the photon number and carrier density, as shown in equations (S23a) and (S23b). By transforming $\tilde{\omega} \to -\tilde{\omega}^*$ and taking the complex conjugate of equation (S27) we obtain another equation:

$$[1 - \mathscr{G}^*(-\tilde{\omega}^*) + B^*(-\tilde{\omega}^*)]\, \delta E^*(-\tilde{\omega}^*) + B^*(-\tilde{\omega}^*)\delta E(\tilde{\omega}) = F^*(-\tilde{\omega}^*). \tag{S29}$$

Equations (S27) and (S29) can be compressed into matrix form,

$$\begin{pmatrix} 1 - \mathscr{G}(\tilde{\omega}) + B(\tilde{\omega}) & B(\tilde{\omega}) \\ B^*(-\tilde{\omega}^*) & 1 - \mathscr{G}^*(-\tilde{\omega}^*) + B^*(-\tilde{\omega}^*) \end{pmatrix} \begin{pmatrix} \delta E(\tilde{\omega}) \\ \delta E^*(-\tilde{\omega}^*) \end{pmatrix} = \begin{pmatrix} F(\tilde{\omega}) \\ F^*(-\tilde{\omega}^*) \end{pmatrix}; \tag{S30}$$

their solutions can be written as

$$\delta E(\tilde{\omega}) = \frac{F(\tilde{\omega})(1 - \mathscr{G}^*(-\tilde{\omega}^*) + B^*(-\tilde{\omega}^*)) - F^*(-\tilde{\omega}^*)B(\tilde{\omega})}{D(\tilde{\omega})}, \tag{S31a}$$

$$\delta E(-\tilde{\omega}^*) = \frac{F^*(-\tilde{\omega}^*)(1 - \mathscr{G}(\tilde{\omega}) + B(\tilde{\omega})) - F(\tilde{\omega})B^*(-\tilde{\omega}^*)}{D(\tilde{\omega})}, \tag{S31b}$$

where we defined the system determinant:

$$D(\tilde{\omega}) = (1 - \mathscr{G}(\tilde{\omega}) + B(\tilde{\omega}))(1 - \mathscr{G}^*(-\tilde{\omega}^*) + B^*(-\tilde{\omega}^*)) - B(\tilde{\omega})B^*(-\tilde{\omega}^*) \tag{S32}$$

The oscillating solutions are the sidemodes of the lasing mode, and they are found by solving the zeros of $D(\tilde{\omega})$, where the solution with $\tilde{\omega} = 0$ represents the lasing mode itself. If the sidemodes have positive imaginary part of $\tilde{\omega}$ then their oscillations are damped and the lasing mode is stable. On the contrary if the imaginary part of $\tilde{\omega}$ is negative then the lasing mode is unstable. Along with the sidemodes solutions, the model can be used to estimate the relative intensity noise spectrum $RIN(\tilde{\omega})$. For this task we don't need the frequency domain to be extended over the complex plane since we are interested in the stationary spectra, which can be represented in the real frequency domain. Following the procedure illustrated by Detoma,[1] we can define the time domain expression of the total field $E_{laser}(t)$ as

$$E_{laser}(t) = E_s(1 + \rho(t))e^{i(\omega_s t + \phi(t))} \simeq E_s(1 + \rho(t) + i\phi(t))e^{i\omega_s t}. \tag{S33}$$

The envelope time domain fluctuation is then $\delta E(t) = E_s(\rho(t) + i\phi(t))$, and its Fourier transform is

$$\delta E(\tilde{\omega}) = E_s(\rho(\tilde{\omega}) + i\phi(\tilde{\omega})); \tag{S34}$$

The amplitude and phase fluctuations of the envelope field can now be expressed as function of $\delta E(\tilde{\omega})$ and $\delta E^*(-\tilde{\omega})$:

$$\rho(\tilde{\omega}) = \frac{1}{2E_s}(\delta E(\tilde{\omega}) + \delta E^*(-\tilde{\omega})), \tag{S35a}$$

$$\phi(\tilde{\omega}) = \frac{1}{2iE_s}(\delta E(\tilde{\omega}) - \delta E^*(-\tilde{\omega})). \tag{S35b}$$

The relative intensity noise spectrum can be defined by means of the autocorrelation of the field amplitude fluctuations by using the Wiener-Khinchin theorem and substituting equations (S31a) and (S31b) into (S35a). In addition to the usual terms, such as $F(\tilde{\omega})F^*(-\tilde{\omega})$, the square modulus of $\rho(\tilde{\omega})$ brings other terms dependent from $F_I(\tilde{\omega})F_I(-\tilde{\omega})$. By numerical inspection we found that, regardless of the parameters employed, $\lim_{T\to\infty}[1/T\langle F_I(\tilde{\omega})F_I(-\tilde{\omega})\rangle] = 0$. An intuitive explanation can be given: unlike the case of the autocorrelation function $F_I(\tilde{\omega})F_I^*(-\tilde{\omega})$, the random phases of $F_I(\tilde{\omega})$ and $F_I(-\tilde{\omega})$ are summed up instead of being subtracted. For each $\tilde{\omega}$, the total phase of $F_I(\tilde{\omega})F_I(-\tilde{\omega})$ is then randomly distributed, even for $\tilde{\omega} = 0$. Now the square modulus of $\rho(\tilde{\omega})$ can be easily expanded, and the following expression for $RIN(\tilde{\omega})$ is obtained:

$$\begin{aligned} RIN(\tilde{\omega}) &= \lim_{T\to\infty}\frac{1}{T}\frac{\left\langle|\delta P(\tilde{\omega})|^2\right\rangle}{P_s^2} = 4\lim_{T\to\infty}\frac{1}{T}\left\langle|\rho(\tilde{\omega})|^2\right\rangle = \\ &= \lim_{T\to\infty}\left(\frac{|1-\mathscr{G}^*(-\tilde{\omega})|^2}{|D(\tilde{\omega})|^2}\langle F(\tilde{\omega})F^*(\tilde{\omega})\rangle + \frac{|1-\mathscr{G}(\tilde{\omega})|^2}{|D(\tilde{\omega})|^2}\langle F(-\tilde{\omega})F^*(-\tilde{\omega})\rangle\right). \end{aligned} \tag{S36}$$

As we will see in the following, equation (S36), together with equations (S3) and (S10), is useful for calculating the displacement sensitivity of our experimental setup, as given by equation (3) in the main article.

1.3 Displacement sensitivity

The first step to evaluate the system sensitivity is to calculate the photon number as function of the membrane displacement Δx_{theo}, starting from the equilibrium position $L_{ext,0} = 20$ cm. This relation is found by solving equation (S3) for $L_{ext} = L_{ext,0} + \Delta x_{theo}$. As said previously, for continuous variations of Δx_{theo}, also the lasing mode defined by (ω, ΔN, P) satisfying equations (S3) and (S10) varies continuously. When the sidemodes become undamped this condition is not fullfilled anymore because the lasing action becomes unstable.

Using the parameters in Table S1, we can solve equation (S3) for different Δx_{theo} and find the photon number $P(\Delta x_{theo})$ using equation (S10) (if equation (S32) guarantees mode stability). For a fixed I we found again several solutions for the photon number, in analogy to the oscillating modes reported in Fig. S2a. We will see that the lasing one (the one producing the largest photon population for a fixed current) always corresponds to a maximum of the solution curves plotted in Fig. S3a. This point is very close to the instability region and perturbations of the system can lead to mode competition which is unwanted and could produce effects which are not included in our model. To increase the stability region it would be desirable to work in the linear region of the curves plotted in Fig. S3a; to do so, at first lasing action is established to a certain bias when the Littrow cavity laser is isolated from the membrane. As discussed previously the modes are described by a family of points (similar to the ones reported in Fig. S2a), and the central mode remains the one with the lowest threshold gain. After the membrane is coupled (adiabatically) to the lasing cavity the system evolves to the new mode with lowest threshold gain, according to equation

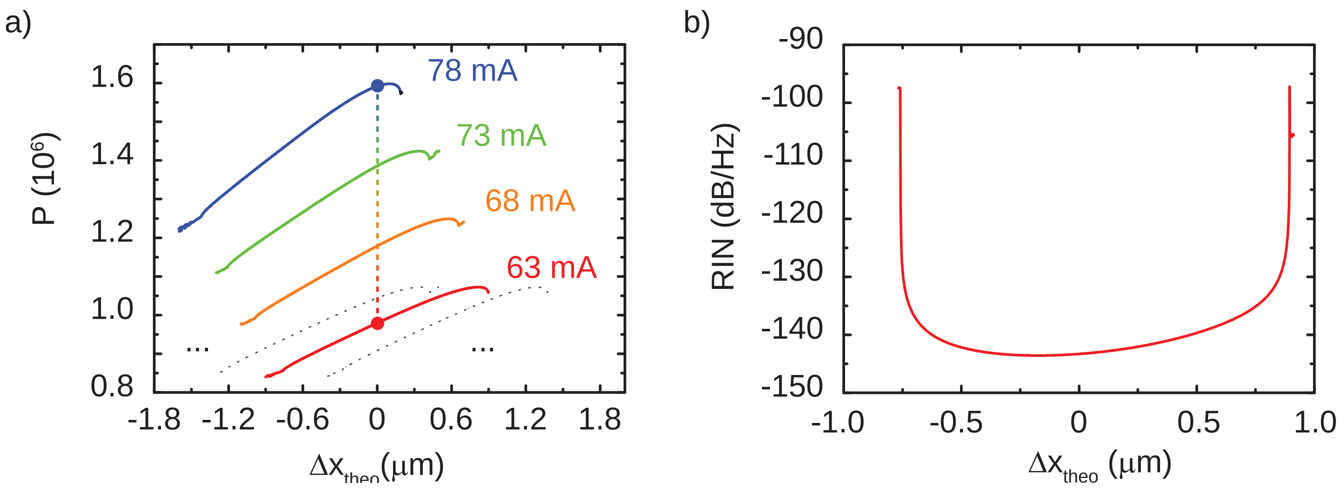

Figure S3. a) Photon sensitivity curve, for different bias currents I. In order to reach the central region of the red curve, the Littrow laser cavity is isolated from the membrane and I is raised to 78 mA, then the laser is coupled to the membrane fixed at $L_{ext,0} = 20$ cm ($\Delta x_{theo} = 0$). The lasing mode is then found near the right most part of the blue curve, marked by the blue circle. By decreasing I continuously and slowly down to 63 mA, the lasing mode evolves following the dashed line in figure. These modes are stable in the sense of equation (S32), therefore the parameters ω_s and N_s also change continuously, according to equation (S3). At $I = 63$ mA (red curve) the lasing mode is found in the central region. Even if it has not the lowest threshold gain, accessing other modes (indicated by the dotted black curves) would require a mode hop, which is not the case of our lasing mode evolving continuously through stable states. The full dynamic range of the curve can thus be used, exploiting an almost linear relation between Δx_{theo} and P. b) Relative intensity noise (RIN) of the lasing mode at $I = 63$ mA as function of Δx_{theo}, taken at frequency $\tilde{\omega} = 2\pi \cdot 74$ kHz. The RIN is almost flat within a range of few kHz around 74 kHz. When the lasing mode approaches the instability region the RIN drastically increases.

(S3); this is always localized near the rightmost region of the photon sensitivity curve. To go back to the central, almost linear region we can modify I; note that this region has the largest stability range and it grants detection of larger displacements, good repeatability and reduced RIN, as shown in Fig. S3b.

To give an example related to our system parameters, let's consider at first the isolated Littrow cavity and a laser drive up to $I = 78$ mA, followed by coupling to the membrane. At this point the system is in the rightmost part of the photon sensitivity curve, shown by the blue circle in Fig. S3a. Then the bias current is decreased slowly, down to 63 mA. By changing I continuously, both ω_s and N_s of the lasing mode change continuously, if the new mode is stable. This is ensured by solving equation (S32) for its sidemodes, and verifying them to be damped for small oscillations. As a result, the lasing mode evolves continuously, following the dashed line in figure, and the photon sensitivity curve evolves accordingly from the blue one to the red one. The final point, marked by a red circle in figure, is our working point for measuring the membrane displacement. This is not the mode with lowest threshold gain, which belongs to the dashed black curve on the left side; however, only the red curve can be accessed by following the path we described, because the lasing mode evolves through stable states, while getting to the dashed curves would require a mode hop. Therefore, we take the red curve in Fig. S3a as our photon sensitivity curve.

As explained in the main article section "Experimental Setup", we are able to measure the output voltage produced by a photodiode integrated with the laser chip, which gives a readout voltage linearly proportional to the power emitted from the diode facet 1. Therefore, the last ingredient needed to estimate the displacement sensitivity of our compound cavity is a relation between the measured readout voltage, $V_0 + \Delta V(\Delta x_{theo})$, and the photon number, $P = P_0 + \Delta P(\Delta x_{theo})$, where V_0 and P_0 are the reference readout voltage and photon number (for $\Delta x_{theo} = 0$), whereas ΔV and ΔP are their respective variations. In order to convert the photon number found from equation (S10) into the readout voltage, we assume a linear relation, $\mathbb{W} = a_1 P$, between the output power $\mathbb{W}$ and P, when the lasing mode is stable. We found that also the total voltage measured by our photodiode varies linearly with $\mathbb{W}$, say $V = a_2\mathbb{W}$. Clearly, we have both $\Delta V = a_1 a_2 \Delta P$ and $V_0 = a_1 a_2 P_0$. Therefore, a_1 and a_2 can be simplified to find

$$\frac{\Delta V}{V_0} = \frac{\Delta P}{P_0}. \tag{S37}$$

Thanks to equation (S37), the photon number can be converted to the readout voltage. In our case we have $V_0 = 5.5$ mV, and $P_0 = 9.784 \cdot 10^5$, as found from the $(\Delta x_{theo}, P)$ curve, the red one in Fig. S3a. If the analysis is restricted to the interval $\Delta x_{theo} \in [-500\,\text{nm}, 500\,\text{nm}]$, by linear fit we find

$$\Delta x_{theo} = 1.212 \pm 0.001 [\text{pm} \cdot \text{nV}^{-1}] \Delta V. \tag{S38}$$

At the same time we can estimate the fundamental measurement imprecision, associated with the photon number fluctuations, and then to the RIN. In order to give a rough overestimate, we may take $RIN \sim$ constant $= -140$ dB/Hz. Using equation (S37) the imprecision $\delta V(f)$ is found, where f is the analyzed frequency:

$$\delta V \sim V_0 \sqrt{RIN} = 0.550 \frac{\text{nV}}{\sqrt{\text{Hz}}}. \tag{S39}$$

Equations (S38) and (S39) can be employed to find the imprecision on the displacement, $\delta x_{theo}(f)$:

$$\delta x_{theo}(f) \sim 0.667 \frac{\text{pm}}{\sqrt{Hz}}. \tag{S40}$$

Now we are going to find the total imprecision for the displacement measurement integrated over the spectrum. The displacement spectral density of the membrane is well fitted by a Lorentzian function

centered on the resonance frequency, therefore we may assume that most of the membrane mechanical energy is stored within a spectral window as large as four times the resonance bandwidth (Θ) , centered on the resonance frequency. The total displacement imprecision associated with the compound cavity is

$$\delta x_{cc} = \sqrt{(0.001[\mathrm{pm}\cdot\mathrm{nV}^{-1}]\Delta V)^2 + (0.667\,\frac{\mathrm{pm}}{\sqrt{Hz}})^2 \times 4\Theta}. \tag{S41}$$

As an example, we calculate the total imprecision associated with the measurement of the membrane thermal motion reported in the main text, where the resonance linewidth is ~ 2 Hz. δx_{cc} gives an estimate of the imprecision associated with the compound cavity, without accounting for the noise brought by the measurement apparatus. In the experiment we found this contribution to be a flat spectrum noise (considering a few kHz spectral window), with amplitude on the order of $\delta V_{\mathrm{detection}}(f) \sim 2\,\mathrm{nV}/\sqrt{\mathrm{Hz}}$. In the case of thermal noise, we have $\Delta V = 33.6\mathrm{nV}$ producing $\delta x_{cc} = 1.885\,\mathrm{pm}$. By using equation (S38) we obtain a total RMS displacement imprecision of $\sqrt{(\delta x_{cc})^2 + (\delta x_{\mathrm{detection}})^2} = 4.790$ pm (we assumed the two imprecision sources to be uncorrelated). In the case of the experimental measurement we have to add also the imprecision due to the calibration, which is described by equation (4) in the main text, and is reported here for convenience:

$$\Delta x_{cal} = 2.6 \pm 0.3\,\frac{\mathrm{pm}}{\mathrm{nV}}\,\Delta V\,. \tag{S42}$$

In the case of thermal noise the RMS displacement imprecision is $\delta x_{cal} = 10$ pm. By assuming the imprecision to be totally uncorrelated, the final imprecision is

$$\delta x_{\mathbb{T}} = \sqrt{(\delta x_{cc})^2 + (\delta x_{detection})^2 + (\delta x_{cal})^2} \simeq 11\,\mathrm{pm}\,. \tag{S43}$$

Using equations (S42) and (S43), we finally recover the experimental RMS thermal motion of $\Delta x_{\mathbb{T}} = 87 \pm 11$ pm, as reported in the main article.

2 Forced harmonic oscillator with flat spectrum displacement

The total membrane displacement for different environmental pressures (as found by integrating the measured power spectral density of the laser output power and applying equation (S42)) has been analyzed and reported in Fig. 2 in the main text. Here we report a more detailed modeling of this effect by considering our membrane as a one dimensional forced harmonic oscillator. The membrane fundamental vibrational mode is well separated in frequency from higher order modes, and its modal shape is sketched in Fig. S4a. The equation of motion reads as;

$$\ddot{x}(t) = \Delta\ddot{x}_D(t) - \omega_0^2 x(t) - 2\pi\gamma_{tot}\dot{x}(t)\,; \tag{S44}$$

Where the external drive is given by a piezoelectric actuator, whose effect can be modeled by considering a displacement with flat spectrum amplitude $\Delta x_D(f) = 2.6$ pm (extracted from the calibration in equation (S42), for a piezo excitation voltage $V_D(f) = 10$ mV), constant for all the frequencies f in our measurement spectral window. Some of the oscillator parameters, such as the damping rate γ_{tot} and resonant frequency f_0 are taken from the experimental data. With straightforward algebra the displacement spectrum is found in the frequency domain:

$$\hat{x}(f) = -\frac{f^2}{(f_0^2 - f^2) - i\gamma_{tot} f}\Delta x_D(f)\,. \tag{S45}$$

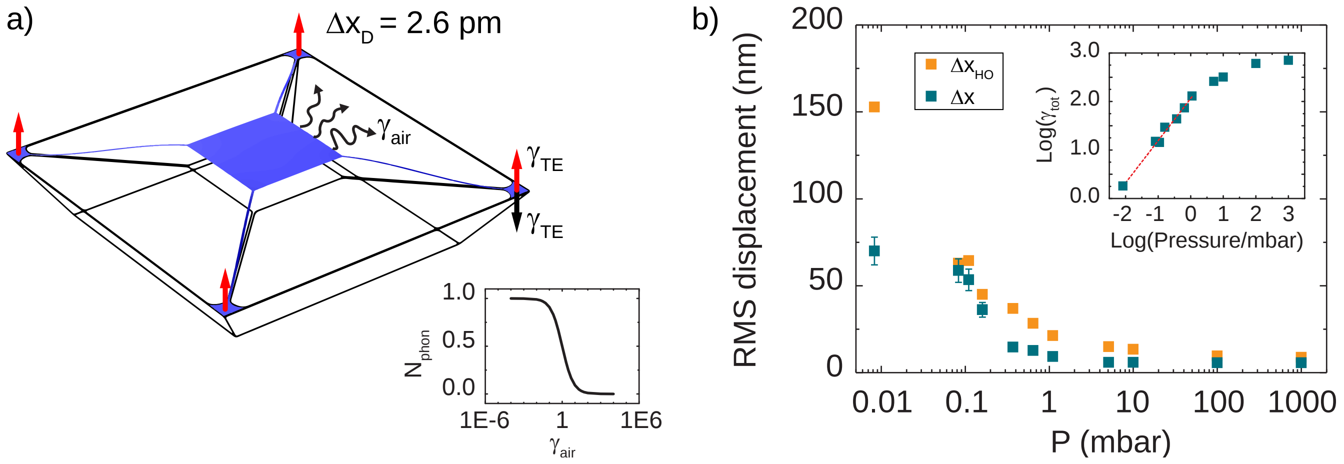

Figure S4. a) Simulated fundamental vibrational mode of the membrane. The system can be seen as a simple harmonic oscillator with two damping channels featured by air friction with damping rate γ_{air} and thermoelastic damping with γ_{TE}. The piezo excitation injects phonons in the system (see the red arrows in figure) from the four tethers, which move integrally with the piezo surface. As the pressure increases, γ_{TE} and the piezo action are almost unaffected by the additional air load, while the total dissipation rate increases due to increasing air friction, resulting in a phonon population decrease like shown in the lower inset. b) RMS displacement extracted from measurements (blue squares) compared to the prediction made from a simple harmonic oscillator forced with a constant displacement (orange squares). The theoretical values are calculated according to equation (S46), by taking the peak parameters from the experimental results; the experimental linewidths are also reported in the upper inset. Note that the disagreement at the lowest pressures is likely due to anharmonic effects due to the large deformations suffered by the tethers.

By using the Wiener-Khinchin theorem, the Displacement Spectral Density $DSD(f)$ is given by the square modulus of equation (S45). The total RMS displacement of the membrane is found by integrating over all the frequencies spanned in our measurements (in our case from 71 kHz to 76 kHz):

$$\Delta x_{HO} = \int_{span} |\hat{x}(f)|^2 df\,. \tag{S46}$$

Figure S4b shows a comparison of the measured total displacement with the one theoretically estimated with this simple model. As can be seen, theory and experiment fit well for most investigated pressures; on the other hand at the lowest pressure, a disagreement between experimental and theoretical data is found. By increasing the vacuum level, the RMS displacement is likely limited by large deformation nonlinearities, which are not included in our linear model; while this is enough to get an amplitude saturation the linewidth, (which is the parameter that enters in the model), is basically unaffected and is still monotonically decreasing, as can be seen from the inset of Fig. S4b. This fact produces the discrepancy, since $\Delta x_{HO} \propto 1/\sqrt{\gamma_{tot}}$. We can safely conclude that, both theoretically and experimentally, the RMS displacement decreases with increasing pressure in the displacement regime where the oscillator is still linear. This can be qualitatively understood also within a rate equation approach. Looking at the picture in Fig. S4a, the membrane can be sketched as a harmonic oscillator populated by N_{phon} phonons, with two damping channels, one originated by air friction with damping rate γ_{air}, and one coming from thermoelastic damping experienced by the four tethers,[9] with damping rate γ_{TE}. The piezo driving the membrane can be seen as a phonon pump (see the red arrows in figure), injecting phonons from the four tethers which move integrally with the piezo surface. Therefore, these phonons are coupled to the membrane mechanical mode with the same rate γ_{TE}. These considerations can be summed up into a simple rate equation:

$$\frac{dN_{phon}}{dt} = \gamma_{TE} Q - (\gamma_{TE} + \gamma_{air}) N_{phon}\,, \tag{S47}$$

whose steady state solution is

$$N_{phon} = \frac{\gamma_{TE}}{\gamma_{TE} + \gamma_{air}} Q\,. \tag{S48}$$

As pressure increases, γ_{air} increases as well, but γ_{TE} remains almost constant. This is because the air load on the piezo actuator is much smaller then the piezo load, so that piezo action is almost the same for all the pressures spanned in the experiment. If the driving voltage is kept the same then the pump rate is constant, whereas the total dissipation rate increases with increasing pressure, resulting in a phonon population decrease like shown in the lower inset of Fig. S4.

3 Radiation pressure and laser heating effects

At odds with our piezo-shook induced vibrations, the standard routine in optomechanical systems is to use the momentum transfer of photon to control the mechanical state of nano-objects.[10] We tested radiation pressure induced heating of the mechanical motion by modulating the lasing radiation at the mechanical frequency through an electro-optic modulator. We verified that to have an impact on the mechanical state, more than 40% peak to peak modulation of laser intensity was necessary, allowing us to completely disregard radiation pressure effects in the reported experiments, since the laser is operating in CW and the self modulations induced by the mechanical feedback are several orders of magnitude smaller than the ones we need to observe any effect. Further investigations of radiation pressure induced dynamical

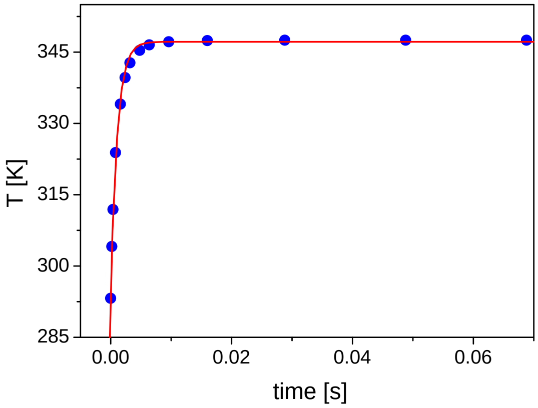

Figure S5. Simulated time evolution of membrane temperature after opening the laser beam. The red line is the best exponential fit, holding a characteristic time of 1.1 ms.

action (such as feedback cooling or heating) are out of the scope of this paper and will be object of future investigations. We further analyzed the impact of thermo-mechanical effects on the membrane; in fact, the temperature rise induces structural changes which could in principle modify the mechanical motion. In our case, finite-element simulations shows a temperature increase in the membrane of roughly 55 K, when we consider the lasing power of the experiment reported in the main article. Most importantly, as shown in Fig. S5, the temperature rise time after the laser beam is shined into the membrane has a very slow dynamics ($\tau_{heating} = 1.1$ ms) and therefore can be considered constant when investigating the two orders of magnitude faster motion of the mechanical mode. Note that even in this case, since the laser is operated in CW, we can safely assume a constant equilibrium temperature which at most could produce a static shift of the mechanical properties of the membrane.

References

1. Detoma, E., Tromborg, B. & Montrosset, I. The complex way to laser diode spectra: example of an external cavity laser strong optical feedback. *IEEE J. Quantum Elect.* **41**, 171–182 (2005).
2. Tromborg, B., Mørk, J. & Velichansky, V. On mode coupling and low-frequency fluctuations in external-cavity laser diodes. *Quantum Semicl. Opt.* **9**, 831 (1997).
3. Van Tartwijk, G. & Lenstra, D. Semiconductor lasers with optical injection and feedback. *Quantum and Semiclassical Optics: Journal of the European Optical Society Part B* **7**, 87 (1995).
4. Levine, A., Van Tartwijk, G., Lenstra, D. & Erneux, T. Diode lasers with optical feedback: Stability of the maximum gain mode. *Physical Review A* **52**, R3436 (1995).
5. Uchida, A. *Optical Communication with Chaotic Lasers: Applications of Nonlinear Dynamics and Synchronization*. Optical Communication with Chaotic Lasers: Applications of Nonlinear Dynamics and Synchronization (Wiley, 2012). URL https://books.google.it/books?id=5eFVoxbcvWYC.
6. Acket, G. A., Lenstra, D., Den Boef, A. J. & Verbeek, B. H. The influence of feedback intensity on longitudinal mode properties and optical noise in index-guided semiconductor lasers. *Quantum Electronics, IEEE Journal of* **20**, 1163–1169 (1984).

7. Agrawal, G. P. & Roy, R. Effect of injection-current fluctuations on the spectral linewidth of semiconductor lasers. *Physical Review A* **37**, 2495 (1988).

8. Burkett, W. H., Lü, B. & Xiao, M. Influence of injection-current noise on the spectral characteristics of semiconductor lasers. *Quantum Electronics, IEEE Journal of* **33**, 2111–2118 (1997).

9. Schmid, S., Jensen, K., Nielsen, K. & Boisen, A. Damping mechanisms in high-q micro and nanomechanical string resonators. *Phys. Rev. B* **84**, 165307 (2011).

10. Aspelmeyer, M., Kippenberg, T. & Marquardt, F. Cavity optomechanics. *Rev. Mod. Phys.* **86**, 1391 (2014).

Table S1. Parameters employed to solve the theoretical laser equations in section 1. The notation, the value and a brief description of the parameters are reported in the three columns.

Parameter	Value	Description
α	4	linewidth enhancement factor
c_0	$3 \cdot 10^{10}\,\mathrm{cm \cdot s^{-1}}$	speed of light in vacuum
q	$-1.6 \cdot 10^{-19}\,\mathrm{C}$	carrier elementary charge
τ_s	$2.08\,\mathrm{ns}$	carrier lifetime
v_g	$9.4 \cdot 10^{9}\,\mathrm{cm \cdot s^{-1}}$	active region group velocity
I_{th}	$33\,\mathrm{mA}$	threshold current
I	$63\,\mathrm{mA}$	current bias
τ_{in}	$16\,\mathrm{ps}$	active region round-trip time
L_D	$\tau_{in} v_g/2$	active region length
V_c	$2.15 \cdot 10^{-10}\,\mathrm{cm^3}$	volume of the active region
N_{th}	$\tau_s I_{th}/qV_c$	threshold carrier density
a_{in}	$9\,\mathrm{cm^{-1}}$	active region internal losses
g_n	$3.2 \cdot 10^{-16}\,\mathrm{cm^2}$	active region differential gain
g_p	$-3.6 \cdot 10^{-6}\,\mathrm{cm^{-1}}$	active region gain saturation factor
g_0	$a_{in} - 1/L_D \log(\lvert r_1\rvert \lvert r_m\rvert)$	threshold gain
γ	$v_g g$	gain rate
G_n	$v_g g_n/V_c$	differential gain rate
G_p	$v_g g_p$	gain saturation rate
r_1	0.55	diode right facet reflectivity
r_2	0.07	diode left facet reflectivity
r_g	0.8	grating maximum reflectivity
$\Delta\omega$	$\pi \cdot 50 \cdot 10^{9}\,\mathrm{rad \cdot s^{-1}}$	grating spectral linewidth
ω_G	$2\pi \cdot 317.46 \cdot 10^{12}\,\mathrm{rad \cdot s^{-1}}$	grating central angular frequency
r_G	$r_g/(1 + j(\omega - \omega_G)/\Delta\omega)$	grating approximated reflectivity
L	$1\,\mathrm{cm}$	grating distance from diode left facet
τ	$2L/c_0$	grating to left diode facet round-trip time
r_{ext}	0.3	Si_3N_4 membrane reflectivity
L_{ext}	$20\,\mathrm{cm}$	Si_3N_4 membrane distance from diode left facet
τ_{ext}	$2L_{ext}/c_0$	Si_3N_4 membrane to diode left facet round-trip time
